# Non-Genetically Improving the Natural Cytotoxicity of Natural Killer (NK) Cells

**DOI:** 10.3389/fimmu.2019.03026

**Published:** 2020-01-13

**Authors:** Martin Villalba, Catherine Alexia, Anais Bellin-Robert, Alexis Fayd'herbe de Maudave, Delphine Gitenay

**Affiliations:** ^1^IRMB, Univ Montpellier, INSERM, CHU Montpellier, CNRS, Montpellier, France; ^2^IRMB, CHU Montpellier, Montpellier, France; ^3^IRMB, Univ Montpellier, INSERM, CHU Montpellier, Montpellier, France

**Keywords:** NK cells, microenvironment, monoclonal antibodies (mAbs), antibody-dependent cell cytotoxicity (ADCC), autoimmune diseases, CD45RARO

## Abstract

The innate lymphocyte lineage natural killer (NK) is now the target of multiple clinical applications, although none has received an agreement from any regulatory agency yet. Transplant of naïve NK cells has not proven efficient enough in the vast majority of clinical trials. Hence, new protocols wish to improve their medical use by producing them from stem cells and/or modifying them by genetic engineering. These techniques have given interesting results but these improvements often hide that natural killers are mainly that: natural. We discuss here different ways to take advantage of NK physiology to improve their clinical activity without the need of additional modifications except for *in vitro* activation and expansion and allograft in patients. Some of these tactics include combination with monoclonal antibodies (mAb), drugs that change metabolism and engraftment of specific NK subsets with particular activity. Finally, we propose to use specific NK cell subsets found in certain patients that show increase activity against a specific disease, including the use of NK cells derived from patients.

## Introduction

Innate lymphoid cells (ILCs) play a main role in immune-related disorders and are divided into three groups: ILC1s, ILC2s, and ILC3s (1). Natural killer (NK) cells, which belongs to the ILC1 group, are bone marrow derived cytotoxic lymphocytes (CL) that are well-equipped for the destruction of target cells without the need for prior antigen stimulation. In peripheral blood, human NK cells are mostly CD3^−^CD56^dim^ cells with high cytotoxic activity, while CD3^−^CD56^brigth^ cells excel in cytokine production (2). Additional markers can be used to identify specific subsets within these NK cell populations (2–4). *In vitro* evidence indicates that CD56^bright^ NK cells are precursors of CD56^dim^ NK cells and this might also be the case *in vivo* (3). In contrast to T cells, grafted NK cells show short live, low expansion and low alloreactivity such as graft-versus-host (GVH) in humans. Hence, NK can provide a potential source of allogeneic “off-the-shelf” cellular therapy and mediate major anti-target effects without inducing potentially lethal alloreactivity. Given the multiple unique advantages of NK cells, researchers are now exploring different ways to expand and/or activate them for clinical purposes.

## NK Cells in Clinics: the Problems

Researchers working on the clinical use of NK cells have found numerous challenges. First, this cell lineage represents a low percentage of lymphocytes, usually estimated to 5–15%. In addition this changes during human development (4), making the transfer of sufficient allogeneic cells from a single donor to a patient challenging.

Second, NK cells have low lifespans, in average 1 week (5), suggesting that allogenic cells will shortly survive after engraftment. However, these results should be taken with caution. Lifetime studies were performed using deuterium incorporation, and only actively dividing cells incorporate it. Hence, this technique may not account for long-lived, non-dividing cells. Moreover, researchers normally focus on peripheral blood, hence NK cells mainly homing in lymph nodes such as CD56^bright^ cells are not taken into account in their real weight (5). But, studies in blood are valid considering that allogeneic NK cells for engraftment are obtained from peripheral blood. Moreover, *in vitro* stimulated NK cells normally gain a mature phenotype despite high CD56 expression (6). Therefore, the previous estimates are a reasonable proxy for the amount of time NK cells will be active after allogenic engraftment. In agreement, the persistence of *ex vivo* haploidentical IL-2-activated and -expanded NK cells ranges between 7 and 10 days in patients with AML, NHL, and ovarian cancer (7).

The third challenge is that NK cells show doubling times of 1.25 days after activation (8). This is significantly longer than T cell doubling time during the initial expansion phase, which are 8 and 11 h for CD8^+^ and CD4^+^ T cells, respectively (9). Moreover, after allogeneic engraftment most clinical results failed to show significant expansion of donor NK cells (6, 7, 10–13). Perhaps the high renew and short lifespan account for these poor *in vivo* expansions because NK cells have already strongly expanded during their maturation and they are prone to “effector-like” phenotype, at least in the blood population.

Fourth, naïve NK cells possess a relatively low activity compare to activated cells (6, 14). This could be responsible of the low efficacy of NK cell-mediated therapies (11–13).

Fifth, there are several attempts to activate endogenous NK cells, e.g., by blocking NK cell inhibitory receptors. This led to the development of IPH2101, a killer inhibitory receptors (KIRs)/KIRL blocking antibody (Ab) (15), or monalizumab, a humanized anti-NKG2A Ab (16). This approach has the inconvenience that in cancer patients NK cells are hyporeactive (11, 12, 17). Moreover, new therapies such as NK cell-based therapies are usually tested on patients with advance clinical stages, which correlate with enhance NK cell dysfunction, at least in multiple myeloma (18). This suggests that endogenous NK could be unable to eliminate tumor cells even after releasing KIR inhibition. Interestingly, recent clinical data also in myeloma suggest that such antibodies can modify the endogenous NK repertoire and make them further hyporeactive (19). Other clinical attempts to activate endogenous NK cells include the use of lenalidomide [LEN; (20, 21)]. Biological results from the Phase Ib/II clinical trial GALEN suggest that LEN could facilitate obinutuzumab (OBZ)-mediated NK cell activation (21), as was observed with rituximab (RTX) (22). In fact cancer patients, at least those with hematological cancers, already possess NK cells, which recognize and kill tumor cells, but are unable to control the disease (21, 23, 24). Why only a fraction of NK cells is fighting against the tumor is unknown. Which is known is that blood-born cancer cells use different mechanisms for immune escape (25, 26), e.g., by inducing NK cell dysfunction (27). This mechanism has also been observed in a variety of solid tumor patients (17).

Due to all these adverse points recent clinical approaches target *in vitro* expanded and activated NK cells and hence the use of allogeneic NK cells.

## Mechanisms of NK Cell Expansion

In this context, clinical-grade production of allogeneic NK cells is efficient (28) and NK cell–mediated therapy, including the use of *in vitro* expanded allogeneic NK cells, seems safe (11, 13, 28–31). This review does not focus on NK cell expansion, but in how we can “naturally” increase NK activity. There are recent reviews regarding NK cell expansion, e.x. (32).

But, it is important to note that choosing the correct donor can improve the killing activity of NK cells. There are different possibilities to choose the “best” donor including selection od donors with HLA/KIR mismatch with the patient (33), donors with a group B KIR haplotype (these donors have 1 or more of the B-specific genes: KIR2DS1, 2, 3, 5, KIR2DL2, and KIR2DL5) (34) or even donors with KIR2DS2^+^ immunogenotype (35).

New attempts try developing disease-specific cytokine cocktails to activate *in vitro* patient NK cells (36–38). This is pertinent because *in vitro* the effects of these cocktails are different between patients and healthy donors. (39). However, despite the strong cytolytic potential of expanded NK cells against different tumors *in vitro*, clinical results have been very limited (11–13), e.g., NK are considered highly cytotoxic against AML tumor cells, but their efficacy as monotherapy in the clinic is low (11–13). Moreover, the results using NK cell therapy in animal models of solid tumors or in clinical trials are disappointing, even if NK cells can eliminate the engrafted cell type or the primary tumor cells *in vitro* (11–13). In this context, it should be noted that different culture media affect tumor recognition by NK cells (40). In summary, there is not any expansion protocol that produces allogeneic NK cells able to efficiently eliminate solid tumor cells *in vivo*. Why NK cells destroy most targets *in vitro* but not *in vivo* is unknown. Tumor cells strongly modify the expression of ligands, which are recognized by NK cell activating or inhibiting receptors when cultured *in vitro* (40). This could lead to the mistrust that those specific tumor cells would be NK sensitive or resistant *in vivo*. Allogeneic NK cells survive for several days in patient's body (see above), hence their initial survival is probably not the blocking step for their efficacy *in vivo*. Impaired tumor infiltration and/or low cytolytic activity in the immunosuppressive tumor environment are usually pointed out as responsible of their low function *in vivo*. Hence, researchers have focused on protocols to activate them enough to bypass these clinical obstacles.

There are many protocols to expand and activate *in vitro* NK cells (6, 11, 13, 28–31). For many clinical uses, the manufactured cells should express the FcγRIIIa, also called CD16. The probably exception is those protocols wishing to generate chimeric antigen receptor (CAR) NK. We have produced umbilical cord blood (UCB)-derived NK cells because they are rapidly available, present low risk of viral transmission and have less strict requirements for HLA matching and lower risk of GvH disease (GvHD) (11). Expansion was driven by Epstein–Barr virus (EBV)-transformed lymphoblastoid B cell lines as accessory cells, which induce a unique NK cell genetic reprogramming (14), generating effectors that overcome the anti-apoptotic mechanism of leukemic cells (41) and that are able to eliminate tumor cells from patients with poor prognosis (42). NK cells obtained with this protocol perform antibody-dependent cell cytotoxicity (ADCC) *in vitro* and *in vivo* with different therapeutic antibodies and against diverse target cells (6).

NK cell expansion is extremely challenging from an industrial point of view (43, 44), partly due to the problems described in the previous section. In addition, NK cell production should be easily scaled up and developed with good manufacturing practices (GMP). Several biotech companies are now producing NK cell-based products that could reach the clinic in the future (44). We will discuss now mechanisms to naturally improve NK cytotoxicity. We will not discuss about lympho-depleting chemotherapy, e.g., cyclophosphamide followed by daily fludarabine, which is already largely use in clinics prior to NK cell infusion (45).

## Cytokines Mediate NK Activation

Generally, when NK will reach the target microenvironment they will receive a burst of cytokines from other cells, e.g., those immune cells that have already infiltrated the tumor. These cytokines affect NK cell behavior and activation and has extensively been reviewed elsewhere (46). Hence, we will only briefly describe some few uses. IL-2 and IL-15 are strong NK cell activators, but their clinical use *in vivo* is challenging due to their toxicity (44). Moreover, IL-2 expands and mobilizes regulatory T cells, which dampen the activity of several effector cells including NK (44). IL-15, although less toxic than IL-2, is limited by its short half-life leading to poor functional activity *in vivo*. However, *in vitro* both cytokines are very efficient stimuli to activate and expand NK cells (6, 14). In fact, membrane-bound IL-15 is currently the best activating cytokine (47), although membrane-bound IL-21 is becoming an interesting challenger (48, 49). In any case, long-term cytokine treatment can lead to NK cell exhaustion, which will inhibit NK activity (50).

## Modifying the Target Microenvironment

**Figure 1:**
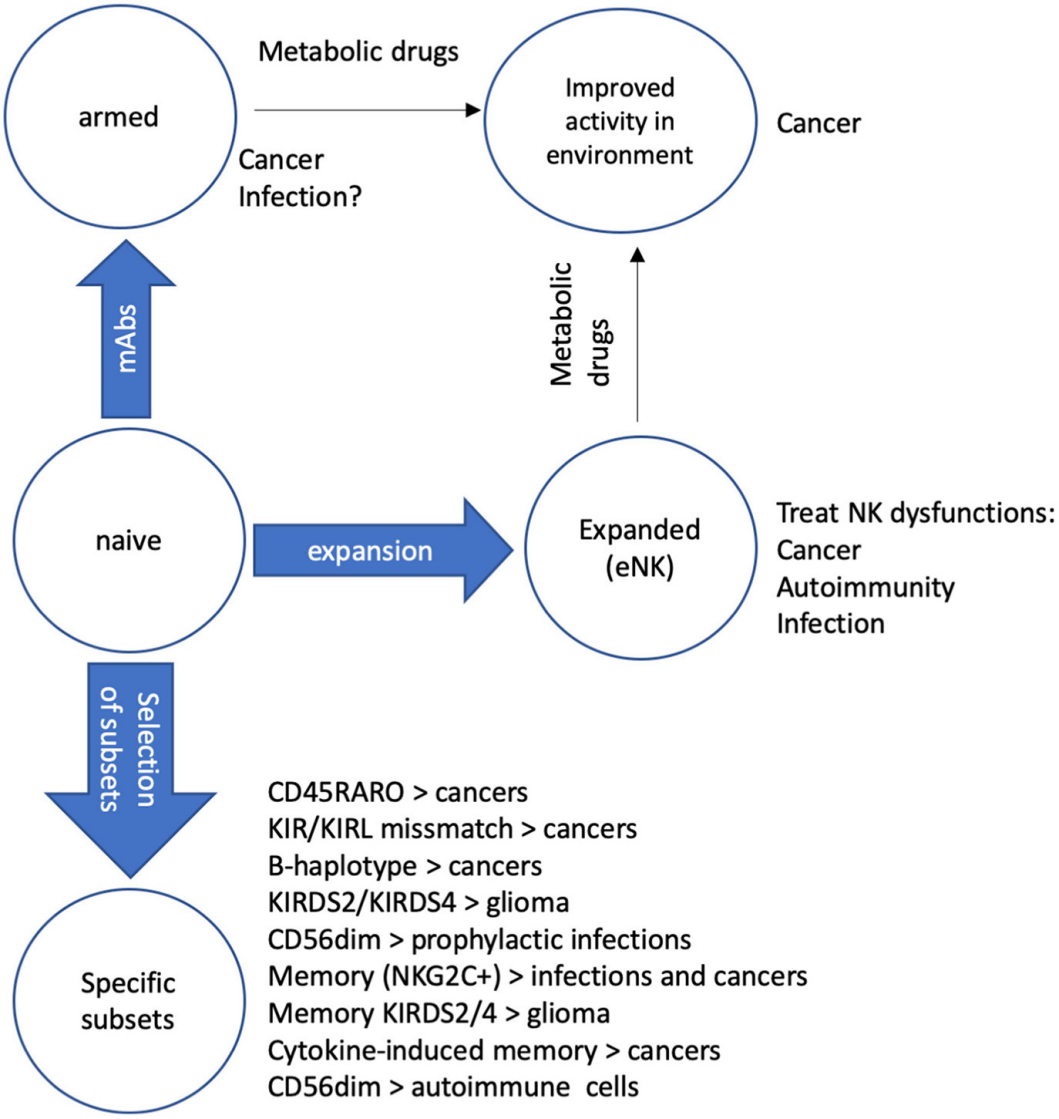
Protocols to recover/improve NK function. We describe several mechanisms to improve NK activity in patients. Naïve NK cells can be “armed” with mAbs that recognize tumor antigens (Ags) to improve their cytolytic activity against cancer cells (6). If specific mAbs against Ags of different pathogens are available, they can be used to arm NK cells to fight infections, mainly in immune compromised patients (54, 55). NK cells can be expanded (eNK) to recover NK cell functions in several diseases such as cancer, autoimmune diseases and infections (32). Treatment of patients with metabolic drugs that modify the microenvironment of the target can increase the function of both “armed” NK and eNK (25, 53). We also believe that it is possible transfer specific NK cell subsets to treat different diseases such as cancers (11–13), including glioblastoma (56) that has a poor prognosis. Some NK subsets, e.x. memory NK cells could also fight infections (57) when engrafted in patients. Finally, in autoimmune diseases could be clinically relevant to replace immature CD56^bright^ NK, which are mostly proinflammatory with mature CD56^dim^ NK, which eliminate activated immune cells. These two NK subsets differentially express various chemokine receptors, which attract them to distinct organs (58, 59). Hence, locally playing with different chemokines should naturally facilitating the recruitment of a specific subset.

Tumor cells, directly or by controlling non-transformed cells, modify the environment to make it immunosuppressive and avoid destruction by effector immune cells (25, 26, 50). We will discuss here some approaches that can reverse this “negative” microenvironment. We will not discuss drugs that *per se* sensitize target cells to NK cells. Tumor-induced modifications include metabolic changes with the production of metabolites that negatively affect NK cell cytotoxicity, e.g., lactate (26, 50). This is the classical metabolite produced by tumor cells under the Warburg effect: cells perform glycolysis even in the presence of ample oxygen (26). To recover the reducing power of NAD^+^, which has been reduced to NADH^+^-H^+^ during glycolysis, cells reduce pyruvate creating lactate. This mechanism recovers the cell reducing power and allows the glycolysis to proceed. During the Warburg effect, the products that are not oxidized, i.e., that are not consumed to produce CO_2_, serve to create new intermediate metabolites that are used for anabolism. But in addition, tumor cells release lactate to the external medium. This acidifies the environment and inhibits the antitumor response of CLs because the killing activity of these cells is extremely sensitive to the decrease in pH (50, 51). There are some compounds such as dichloroacetate (DCA) or metformin that inhibit the Warburg effect and block lactate production (26, 52, 53). It is hence conceivable that such drugs could increase the cytolytic activity of NK, or other CLs, *in vivo* (Figure 1).

In contrast, during the killing of yeast cells or cryptococcoma, NK cells appeared to profit from the acidic pH of the microenvironment by displaying enhanced perforin degranulation and killing capacity (51). Therefore, an interesting possibility would be to modify the NK-tumor environment to match that of NK-yeast cells/cryptococcoma in order to increase NK cytotoxicity even at low pH.

Another way to increase NK activity would be to decrease adenosine concentration in tumor environment. This nucleotide is found as much as 100-fold higher in tumors than in normal tissues and contributes to immune evasion by inhibiting for example NK cell cytolytic activity (50). The ectonucleotidases CD39 and CD73 produce large amounts of adenosine, hence their inhibition decreases tumor growth and metastasis. This type of treatment has reached the clinic with the anti-CD73 antibody Oleclumab (50).

Several vitamins, e.g., A, C, and E, induce changes in NK cell markers associated to activation (60). Vitamin A/retinoic acid increases target expression of natural-killer group 2, member D (NKG2D) ligands in mouse, RAE-1 (60), and humans, MICA/B (61, 62). However, it can activate (60, 62) or inhibit (14, 60, 63) NK activity depending on the cellular context. Hence, their use in clinics must be carefully studied.

## ADCC is Natural: NK Cells and mAbs

Cell-mediated immune defense includes ADCC. NK only harbor the activating Fcγ receptors CD16a and FcγRIIc, also known as CD32c. This gives NK a preponderant role in ADCC in humans (64). Although not include in the so-called “natural NK cytotoxicity,” ADCC is totally a natural physiological process mediated, at least in large part, by NK cells, but involving coordination and crosstalk of different immune cells (64). Through ADCC NK cells can modulate the adaptive immune response and generate long term protection (65).

Differential response to therapeutic mAbs has been reported to correlate with a specific polymorphism in *CD16* (V158F) (66). This polymorphism is associated with differential affinity for mAbs (64). Indeed FcγRs variant play an important role in determining prognosis of monoclonal IgG antibodies (mAbs) therapy (67). Hence, an obvious possibility is using NK cell from donors with the 158V polymorphism, which shows increase affinity for Fc and better prognosis to mAb treatment (64, 67). This engrafted NK should show improved activity after transplantation, mainly when associated to mAb cotherapy. Conversely, different approaches modify the antibody Fc region to increase patient NK cell activity. For example, obinutuzumab, an anti-CD20 mAb, is afucosylated to increase CD16 binding and thereby enhance its ADCC activity (68).

## Arming NK Cells

As previously described NK cells recognize antibody-opsonized target cells and hence take advantage of the exquisite selectivity of mAb to generate a discriminatory immune response against target cells. An interesting possibility of increasing NK function is loading mAbs into the NK CD16 Fc receptor, giving them an exogenous selectivity against target cells (Figure 1). Recent data show that expanded NK retain RTX on their CD16 at least overnight (6). Moreover, RTX-armed NK show improved cytolytic activity compared to non-armed NK cells. In fact, *in vitro* results using RTX and CD20^+^ tumor cells deerived from chronic lymphocyte leukemia (CLL) patients do not show any differences on NK cell-mediated ADCC between opsonizing targets or “arming” NK (6).

There are other possibilities to “arm” expanded NK cells, e.g., (i) with activating receptors that enhance their natural anti-tumor capacity; (ii) with chimeric antigen receptors (CAR) that can redirect them toward specific tumor targets (45); or (iii) with death receptor ligands such as a glycosylated form of TNF-related apoptosis-inducing ligand (TRAIL) fusion protein (69). These armed NK cells show improved antitumor function, but these approaches require genetic modification of NK cells, and we do not consider them “natural.”

## Naturally Occurring Antitumor NK Cells: Trogocytosis and the CD45RARO Paradigm

The NK cell population with antitumor activity has recently been identified (21, 23, 24). In multiple hematological cancer patients there is a population of highly activated CD56^dim^CD16^+^ NK cells that have recently degranulated, evidence of killing activity. These cells generally expressed NKp46, NKG2D, and KIRs, whereas expression of NKG2A and CD94 is diminished. They are also characterized by a high metabolic activity and active proliferation. Notably, these NK cells carry, non-NK, tumor cell antigens on their surface, evidence of trogocytosis during tumor cell killing, i.e., they carry CD19 in B cell-derived cancers and CD14 in myeloid-derived cancers (21, 23, 24). The antitumor NK cells are distinguished by their CD45RA^+^RO^+^ phenotype, as opposed to non-activated cells in patients or in healthy donors displaying a CD45RA^+^RO^−^ phenotype (21, 23, 24). Therefore, antitumor NK cells exist (23). Hence, there is the possibility of selectively expand this population. However, *in vitro* expansion does not really produce similar phenotypes to those found in cancer patients. Moreover, NK cell markers change *in vitro* (23, 24). Another possibility would be to exchange the antitumor population of two cancer patients. Notably, CD45RARO cells show strong activity against a different tumor cell (23). This is reminiscent with previous *in vitro* studies showing that NK cells exhibited enhanced cytotoxicity after a prior co-culture with some tumor cells (70, 71). But the *in vivo* interest of using patient CD45RARO cells to treat other patients goes further that this possible “priming” effect. It is known that tumor cells have been immune sculpted by the host immune system (72). This allows them to immune escape and generate cancers. However, the mechanisms of tumor immune escape are usually different between host/tumor pairs. This suggests that tumors will be better recognized by antitumor NK cells of another patient, supporting the exchange of NK cells between patients (Figure 1). Obviously, the national health agencies should carefully examine this possibility.

Another possibility is transferring NK cell genotypes that show higher activity against a specific cancer, such as the B-haplotype in AML (34) or KIR2DS2 immunogenotype in glioblastoma (35) (Figure 1).

## NK and Infectious Diseases

In contrast to cancer patients, CD45RARO populations have not been described in patients with viral infections yet (23, 24). In view of the safety of allogeneic NK in different tumor treatments described above, their use in infectious diseases is clinically relevant. However, if we usually consider “cancer” as a complex disease, what to say about pathogens so diverse as virus, bacteria, and fungi. Remarkably, a growing body of evidences show that NK cells play a major role in the immunity against all these pathogens, not only by their direct killing of pathogens or infected cells, but also by producing cytokines that activates other immune cells (73). In several pathological conditions leading to immunodeficient patients, allogeneic NK cells could support the recovery of enough protection to decrease infectious complication (54). An example of immunodeficiency occurs during hematopoietic stem cell transplantation (HSCT). The recipient's immune system is usually destroyed with radiation or chemotherapy before the stem cell transfer. Hence, infection is a major complication. Currently, clinicians are trying to use certain immune cell types such as granulocytes or infectious-specific T cells to control infection. Although randomized studies failed to demonstrate a significant survival benefit of granulocyte transfusions after HSCT (73). It is known that rapid reconstitution of NK population protects from both infection (54) and tumor relapse (11, 13, 73). Hence, prophylactic engraftment of NK cells in HSCT patients could protect from infection and decrease relapse, with the advantage that NK cells, and not T cells, target a broad range of pathogens (Figure 1).

If the use of allogeneic NK cells in patients at risk, i.e., immunocompromised patients as those described above, clinically sounds, this is not the case of immunocompetent individuals. Obviously clinical results of allogeneic NK cell engraftment in these patients are lacking. Despite this, we want to discuss certain medical situations in which it could be useful. Again, with the assumption that the transplant would not be toxic to the patient.

First, the severity of influenza can be associated with transient T and NK cell deficiency (55) and with specific haplotypes of killer-immunoglobulin-like receptors (KIRs) (74). Engraftment of NK cells from donors with such haplotypes can improve the prognostic of humans that barely respond to influenza vaccine. A similar approach, i.e., engraftment of allogeneic NK cells, could be clinically relevant to treat patients infected with flavivirus, which includes viruses such as West Nile, yellow fever, dengue, Zika, and Chikungunya. Flavivirus-induced diseases are currently generating major health problems and, interestingly, NK cells play a central role in controlling these viruses (75). In other deadly viral infection such as Ebola, which also decreases peripheral NK cell numbers, the transplant of NK cells could be inefficient because Ebola virus uses specific NK evasion mechanism (76) and can modulate NK function to increase viral pathogenicity (77). The possible use of virus-specific memory NK cells will be discussed below.

Second, NK cells, through release of perforin and granulysin, kill a variety of bacteria including *Mycobacterium tuberculosis, Bacillus anthracis, Escherichia coli, Salmonella typhi*, and *Trypanosoma congolense* (73, 78). NK cells can also eliminate host cells infected with intracellular bacterial pathogens by engagement of target cell death receptors, such as Fas- FasL and TNF-related apoptosis-inducing ligand (TRAIL) (73). The transfer of allogeneic, expanded, NK cells in patient infected with those bacteria and with a bad prognostic could have an obvious clinical benefit.

Third, allogeneic NK cells could also be useful for fungal infections of poor prognosis due to their direct effect against a number of pathogenic fungi including mucormycetes, *Aspergillus fumigatus, Cryptococcus neoformans*, and *Candida albicans*. In addition, NK cells produce a number of cytokines that activate the antifungal activity of other immune cells (73).

## Memory NK Cells

Viral-infected patients have NK subsets that are associated to antiviral immunity and could be used for clinical purposes. Human cytomegalovirus (HCMV) infection promotes expansion of NKG2C^+^ NK cells with memory-like properties (79, 80). Furthermore, NK cells expressing high levels of NKG2C and CD57 are associated with prior HCMV infection. Certain cytokines such as IL-12, which is produced by CD4^+^ monocytes, are mandatory for NKG2C^+^ cell expansion (81). However, there is a lack of evidence concerning their specific effect against HCMV itself or if there is a recall response to HCMV reactivation (82).

These HCMV-specific NK cells can originate from CD16-induced memory-like NK cells and hence they can be waked up by HCMV antibodies (57). Once activated, these cells could not only attack HCMV-infected cells, but also other NK cell targets such as transformed cells. Subsequently, they could be transferred to patients lacking them to generate the desired immunity (Figure 1). Direct transfer of anti-HCMV antibodies would probably not work because these antibodies presumably do not mediate in the initial generation of NKG2C^+^ “adaptive” NK cells in HCMV-seronegative individuals (83).

Most glioblastoma express HCMV proteins and HCMV infection imprint NK cells. In addition, KIR2DS2+ and KIR2DS4+ are more potent killers that bulk NK cells in glioblastoma cells (35). Remarkably, CMV impacts disease progression in glioblastoma and the KIR allele KIR2DS4^*^00101 is an independently prognostic of prolonged survival (56). Hence, the transfer of KIR2DS4^*^00101 NK cells could specifically improve prognosis of glioma patients.

Another possibility is generating “memory”-like NK cells by incubation with different cytokine cocktails, e.g., IL-2/IL-15/IL-18 (57, 82). Some of these cytokines are already part of the current cocktails to amplify and activate human NK cells *in vitro* as described earlier. In fact, several of these protocols also used accessory, target, cells to drive NK cell expansion and/or activation. The target cell contact-dependent priming signals to enhance NK cell activation has already been described, although the priming stimulus is unknown (57). This has not stopped their clinical test in clinics (12).

In summary, exploiting NK cells with memory-like properties might increase the efficacy of these cells and help their clinical development. However, it is uncertain if current protocols to produce *in vitro* expanded NK cells are not really generating “memory-like” NK cells, and hence, the use of “memory-like” NK cell is perhaps already used in clinical studies.

## Recovering NK Activity in Autoimmune Diseases by Replacing Endogenous NK Cells

NK cells from patients of several autoimmune diseases present populations that can contribute to disease progression. In other cases, endogenous NK cells are defective, e.g., in cytotoxicity, due to genetic or environmental facts. Hence, engrafting NK subsets with proper activity could rescue NK activity and improve prognosis. Below we discuss some specific diseases such as rheumatoid arthritis (RA), multiple sclerosis (MS), and systemic lupus erythematosus (SLE). However, similar approaches could also target type I diabetes (T1D) and Sjögren's syndrome (58).

RA patients accumulate immature NK cells in damaged joints. Sinovial fluid (SF) NK (sfNK) cells derived from these patients are enriched in the CD56^bright^ population (84). Moreover, sfNK produce more IFNγ and TNFα after interleukin-15 activation (84, 85). IL-15, which is present in the SF of RA patients, correlates with disease severity and is important in disease progression (59). Hence all this may contribute to the production of proinflammatory cytokines and long-term inflammation (58). The sfNK cell subset, high CD56, CD94/NKG2A, CD69, and NKp44 and low CD16, is unlike any population documented in any other organ and is enriched in patients with erosive deformative RA (DRA) (84, 85). The percentage of total NK cells was doubled in the peripheral blood and tripled in SF of DRA, as compared to non-deformative RA (NDRA), patients (85). Other characteristics of these sfNK in RA are almost absent KIR expression, low CD57 and high natural killer cell p46-related protein (NKp46) (85). Probably the chemokine receptors specifically expressed by immature NK cells facilitate their infiltration into the damaged joints and favor RA damage exacerbation (85). Interestingly, the sfNK CD56^bright^ population express CD16, something that is unique, although its functionality was not investigated (85). Since sfNK may play an important role in destruction of joints, which should implicate their IFNγ and TNFα production, it would be interesting to replace the immature sfNK with mature CD56^dim^ cells (Figure 1). An interesting possibility is using those protocols to produce *in vitro* expanded NK cells described earlier. Although these NK cells present high CD56 levels, they possess all characteristics of mature and activated NK such as KIR and NKG2D expression [e.g., (6)]. The engraft of these cells in damaged joints could reverse the damaging effect of the autologous CD56^bright^ cells.

MS is an autoimmune inflammatory disease affecting the central nervous system (CNS). Autoreactive CD4 T cells targeting myelin components are critical mediators. NK cells can control inflammation by killing activated, autoimmune, T cells (58). Activated T cells increase expression of the death-receptor Fas. In patients in remission, NK highly express Fas ligand (FasL), which can eliminate autoreactive T cells through Fas/FasL interactions (86).

During relapse the FasL^high^ NK population is lost (86). The site of autoimmunity, i.e., the cerebrospinal fluid, is enriched in immature CD56^bright^ NK subset, whereas this population is reduced in peripheral blood (87). Daclizumab, an anti-IL-2Rα antibody, ameliorates CNS lesions with a decrease in blood CD4 T cells and increase in blood CD56^bright^ NK (88). Hence, current knowledge on the biology of MS suggest that engraftment of a mature, cytolytic, CD56^dim^ subset could facilitate elimination of autoreactive T cells (Figure 1). Although a possible negative effect cannot be ruled out due to the presence of NKG2D ligands in oligodendrocytes, astrocytes and microglia (58).

SLE is an autoimmune disorder characterized by production of autoantibodies against DNA and nuclear proteins. Like in RA, there is a polyclonal B-cell activation and expansion. NK cell deficiency correlates with SLE in humans and in mouse models of the disease (58). Again, SLE patients show an increase in the proportion of blood CD56^bright^ NK cells (89). In addition, NK-dependent cytotoxicity decreases (58). Interestingly in pediatric patients, who show the same NK defects (90), the impaired activity is observed at diagnosis (90). Like in previous described autoimmune diseases reconstitution of a mature CD56^dim^ population in SLE patients could improve their prognosis. These approaches requiring the engraftment of “missing” NK cell subsets need proper allogeneic NK recruitment into the target organ. An obvious solution is local engraftment. Another possibility is using a chemokine cocktail. The two NK cell subsets, i.e., CD56^bright^ and CD56^dim^, differentially express various chemokine receptors, which attract them to distinct organs (58, 59). Hence, locally playing with different chemokines should naturally facilitating the recruitment of a specific subset. In anyway, reconstitution of NK cell activity in periphery should improve patient prognosis in these diseases heavily dependent on NK cell function.

## NK Cell Lines: are They Natural?

The difficulties for purify, isolate, expand, and transduce primary NK cells for therapeutic applications led researchers to also focus on NK-cell lines such as NK-92 (NK-92® ATCC® CRL-2407™ and NK-92® MI ATCC® CRL-2408™). There are other NK cell lines, but their antitumor cytotoxicity is questioned (91). In any case, our discussion here on NK-92 cells should be valid for new NK cell lines that could reach the clinic. NK-92 phenotype is CD3^−^CD56^+^CD16^−^ and display cytotoxicity against a wide range of human primary leukemias, e.g., B-ALL and CML and leukemic cell lines *in vitro* and in SCID mouse models (92). Stable expression of mouse and human CD16 gives ADCC to NK-92 cells and generates the cell lines NK-92 ^mCD16^ and NK-92 ^hCD16^, respectively (93). In addition, they are a renewable resource to generate CAR-NK-92 cells. In line with our previous comments we will not discuss about these transduced cells. In contrast, non-modified NK cell lines show therapeutic effect without the need of genetic modifications (91). However, transformed cell lines present worries, such as uncontrolled growth, which require irradiation before infusion into patients. This suppress cell proliferation while, hopefully, maintaining enough cell cytotoxic activity. NK-92 cells have completed phase I trials in cancer patients, e.g., NCT00900809 and NCT00990717. Results show that irradiated NK-92 cells are safe even at very high doses with minimal toxicity in patients with refractory blood cancers (94). In addition, they show clinical benefits with 2 out of 12 patients showing complete response (94).

## Conclusion

In a challenging clinical environment with the arrival of “new” cell-therapy products, NK present several advantages and inconveniences. Their clinical improvement by “natural” means that can easily be accepted by natural agencies will greatly favor their use.

## Author Contributions

All authors were involved in preparing and writing the manuscript.

### Conflict of Interest

The authors declare that the research was conducted in the absence of any commercial or financial relationships that could be construed as a potential conflict of interest.

## Abbreviations

ADCC: antibody-dependent cell-mediated cytotoxicity
AML: acute myeloid leukemia
B-CLL: B-cell chronic lymphocytic leukemia
B-NHL: B-cell non-Hodgkin's lymphoma
BCL: B-cell lymphoma
DLBCL: Diffuse large B-cell lymphoma
EBV: Epstein–Barr virus
EGFR: epidermal growth factor receptor
e-NK: expanded NK cells
FL: follicular lymphoma
GMP: good manufacturing practices
GvHD: graft-versus-host disease
HSCT: hematopoietic stem cell transplantation
LEN: lenalidomide
mAbs: monoclonal antibodies
NCRs: natural cytotoxicity receptors
NK cells: natural killer cells
OBZ: obinutuzumab
PFS: progression-free survival
RTX: rituximab
UCB: umbilical cord blood
UCBT: umbilical cord blood transplantation.
